# Effect of Beclin-1 gene silencing on autophagy and apoptosis of the prostatic hyperplasia epithelial cells

**DOI:** 10.1016/j.clinsp.2022.100076

**Published:** 2022-09-08

**Authors:** Rongfu Liu, Song Zhang, Rui Wan, Jiang Deng, Wei Fang

**Affiliations:** a Department of Urology, Yangpu Hospital, School of Medicine, Tongji University, Shanghai, China; b Department of Urology, The First Affiliated Hospital of Xiamen University, Xiamen, Fujian, China

**Keywords:** Prostatic hyperplasia, Beclin-1 gene, Autophagy, Apoptosis

## Abstract

**Objectives:**

This study aims to explore the effect of silencing Beclin-1 gene on autophagy and apoptosis of Benign Prostatic Hyperplasia (BPH) (BPH-1) cells under the condition of Androgen Deprivation (AD) and Autophagy Inhibition (AI).

**Methods:**

Control group (BPH-1 group), empty carrier group (sh-RNA-BPH-1 group) and Beclin-1 silenced group (sh-Beclin1-BPH-1 group) were set. The Beclin-1 gene silencing efficiency was detected by RT-PCR and Western blot. Autophagic flux was monitored by GFP-LC3 cleavage assay and cell apoptosis was analyzed by flow cytometry. The protein expression levels of LC3, Caspase-3, PARP-1, Bcl-2, and Bax were detected by Western blot.

**Results:**

The transfection of sh-Beclin-1 obviously down-regulated the expression of Beclin-1 at both mRNA and protein levels. Under the conditions of AD and AI, silencing of Beclin-1 restrained the autophagy of BPH-1 cells, as evidenced by a decreased number of autophagosomes and down-regulation of LC3-II protein (*p* < 0.001). The results of flow cytometry showed that the apoptotic rate of sh-Beclin-1 group was elevated significantly compared to the other two groups (*p* < 0.01). Western blot results showed that silencing of Beclin-1 promoted 89 kd fragmentation of PARP-1 (*p* < 0.001) and Caspase-3 activation (*p* < 0.01). Moreover, silencing of Beclin-1 resulted in declined Bcl-2 and augmented Bax protein expression in BPH-1 cells (*p* < 0.01), which ultimately led to a decreased Bcl-2/Bax ratio.

**Conclusions:**

The results indicated that the silencing of Beclin-1 gene hampered autophagy while activating apoptosis in BPH-1 cells. Thus, Beclin-1 may participate in an antagonistic relationship between autophagy and apoptosis in BPH.

## Highlights

• Beclin-1 gene silencing could reduce the autophagy level of BPH-1 cells.
• Beclin-1 gene silencing could promote the apoptosis of BPH-1 cells.
• There is an antagonisc relationship between autophagy and apoptosis in BPH-1 cells.

## Introduction

Benign Prostatic Hyperplasia (BPH) is a progressive disease that frequently occurs in middle-aged men, which can cause lower urinary tract obstruction in severe cases and is an inevitable disease associated with aging.^1^ The prostate gland is an androgen-dependent organ. Androgen deprivation promotes apoptosis in prostatic epithelial cells.^2^ Prescription of selective α-adrenergic receptor blockers and 5-α Reductase Inhibitor (5-ARI) over the past few decades paved the transition of BPH therapy from surgery to drug treatment.^3^ As a mainstream BPH management strategy, 5-ARI reduces the concentration of Dihydrotestosterone (DHT), decreases the volume of the prostate, and alleviates the symptoms of the lower urinary tract.^4^ However, one drawback of the 5-ARI treatment is its elongated treatment period which requires at least three months. Hence, it is necessary to clarify the pathogenesis for a better strategy against BPH.

Autophagy is an evolutionary conservative catabolic process that maintains cellular homeostasis.^5^ Increasing evidence supports that autophagy is closely related to the progression of BPH,^6^^,^^7^ but the underlying mechanism has not yet been fully elucidated. As an important mode of programmed cell death, apoptosis can be triggered by both physiological and pathological stimuli or conditions.^8^ In recent years, the crosstalk between autophagy and apoptosis has been observed to be involved in multiple pathophysiological processes, including those related to hormesis, aging and cancer.^9^ The relationship between autophagy and apoptosis is complex, which exerts the synergistic and/or antagonistic effect between autophagy and apoptosis in different conditions.^10^^,^^11^ Chloroquine induces autophagy and increases apoptosis simultaneously in leukemia cells.^12^ Autophagy and apoptosis of prostate cancer cells are mutually exclusive under Androgen Deprivation (AD).^13^ Such crosstalk has also been reported in BPH in recent years. Liu et al. found that Autophagy Inhibition (AI) could enhance the apoptosis induced by Androgen Deprivation (AD) in human Benign Prostatic Hyperplasia (BPH-1) cells.^14^ In addition, they observed an antagonistic relationship between apoptosis and autophagy after a combination of AD and AI for 24 h, suggesting there was an interaction between apoptosis and autophagy in BPH. Nevertheless, the molecular mechanism of apoptosis and autophagy in BPH induced by AD have not yet been reported.

Beclin-1 gene is located on human chromosome 17q21 and is highly homologous with yeast autophagy gene Atg-6,^15^ which plays a central role in autophagy via PI3KC3 complex or Bcl-2. 16, 17, 18 Multiple studies demonstrated the anti-apoptotic role of Beclin-1 in various settings including nutrient deprivation, chemotherapy, irradiation, immunotherapy as well as hypoxia.^19^ Interestingly, caspases, the crucial mediators of apoptosis, can cleave Beclin-1 during cell apoptosis, thereby destroying its pro-autophagic activity. The C-terminal fragments generated by the cleavage of Beclin-1 translocate to mitochondria and sensitize cells to apoptotic signals, thereby promoting apoptosis.^20^ The aforementioned reports emphasize the role of Beclin-1 in the cross-talk between apoptosis and autophagy. Notably, the cleavage of Beclin-1 by caspase-3 was recently observed in the apoptosis of BPH-1 cells induced by AD.^14^ Therefore, the authors hypothesized that Beclin-1 is an important mediator in the crosstalk between apoptosis and autophagy during the progression of BPH. In the present study, a function of Beclin-1 in the induction of autophagy and alteration of apoptosis-related proteins in BPH was explored by transfecting BPH-1 cells with Beclin-1 specific short hairpin RNA.

## Materials and methods

### Ethics

This study was approved by the Ethics Committee of First Affiliated Hospital of Xiamen University.

### Cell culture and transfection

BPH-1 cells (Sangon Biotech Co. Ltd. Shanghai, China) immortalized but non-transformed human prostate epithelial cell lines,^21^ were provided by the Department of Urology of First Affiliated Hospital of Xiamen University.

Cells cultured in phenol red-free RPMI1640 medium that contained 10% charcoal-stripped fetal bovine serum (Gibco) were used to mimic AD conditions.^22^ Chloroquine (CQ) (Sigma-Aldrich, Darmstadt, Germany) was widely used to inhibit autophagy in many studies.^23^^,^^24^ To simultaneously induced AD and AI in BPH-1 cells, in the BPH-1 group, the cells were cultured in phenol red-free RPMI1640 medium that contained 10% charcoal-stripped fetal bovine serum and 5 × 10^−5^ mmoL/L CQ throughout this study.

To specifically knockdown Beclin-1 in BPH-1 cells, sh-RNA against Beclin-1 (sh-Beclin1: 5’-CCCGTGGAATGGAATGAGATT-3’) synthesized by Genepharma (Shanghai, China) was sub-cloned into PLKO.1-puro plasmids (Addgene, Cambridge, MA, USA) to construct lentiviral vectors that were transfected into BPH-1 cells. In brief, BPH-1cells were seeded in 12 well plates at a density of 1.5 × 10^4^ cells per well. Lentiviral vectors carried sh-Beclin1 were transfected into the cells for 24 h using Lipofectamine 2000 transfection reagent (Invitrogen, Carlsbad, CA, USA). These cells were defined as the sh-Beclin1-BPH-1 group. BPH-1 cells transfected with empty PLKO.1-puro plasmids served as the negative control and were defined as the sh-RNA-BPH-1 group. After transfection, cells were harvested, and transfection efficiency was analyzed by RT-PCR and Western Blot.

### RT-PCR

BPH-1 cells with or without transfections were harvested and subjected to RNA isolation using TRIzol reagent (Invitrogen). Using the cDNA synthesis kit, cDNA was reversely transcribed by cDNA synthesis kit (Bio-Rad Laboratories, Hercules, CA, USA). The primer sequences were as follows: GAPDH (forward: 5′-TCCAGGGGTCTTACTCCTTG-3′; reverse: 5′-TCCAGGGGTCTTACTCCTTG-3′); Beclin-1 (forward:5′-CCATGCAGGTGAGCTTCGT-3′; reverse: 5′-GAATCTGCGAGAGACACCATC-3′). The gene expression was determined by RT-PCR on an ABI 7500 Real-Time PCR system (Applied Biosystems, Carlsbad, CA, USA) with the conditions as below: denaturation at 95°C for 2 min; denaturation at 95°C for 45 s, annealing at 60°C for 1 min, extension at 72°C, for 35 cycles; followed by a final extension step at 72°C for 8 min. The samples were analyzed by 2% agarose gel electrophoresis at 100 V for 45 min.

### Western blot

An equivalent amount of protein from each group of cells was subjected to electrophoresis on diverse concentrations of SDS‑PAGE (8%: PARP-1; 10%: caspase-3, Beclin-1, Bcl-2, and Bax; 12%: LC-3) and transferred onto PVDF membranes. After blocking with 5% skimmed milk, the membranes were incubated with primary antibodies overnight at 4°C, followed by incubation with HRP-conjugated secondary antibodies for 2h at room temperature. Protein bands were visualized on X‑Ray film using an enhanced chemiluminescence system. Densitometry was performed using ImageJ v1.45 software (NIH, Bethesda, MD). The information on all antibodies used in this study is listed in Supplementary Table S1.

### Immunofluorescence staining and confocal microscopy

To monitor the autophagic flux, GFP-LC3 cleavage assay was performed. In brief, GFP-LC3 adenovirus was transfected into BPH-1 cells from different groups using Lipofectamine 2000 reagent after the adenovirus packaging and amplification, adenovirus titer determination, adenovirus optimal Multiplicities Of Infection (MOI) determination. Immunofluorescent images were obtained by a confocal microscope (ZEISS, Germany). The number of GFP-LC3 dots was measured by randomly counting at least 20 cells per group in each experiment.

### Flow cytometry

Cells from each group were collected and transferred to an Eppendorf tube. After adding APC Annexin V and 7-AAD Viability Staining Solution, cells were incubated for 20 min at room temperature in dark. Finally, the percentages of Annexin V positive cells were analyzed with flow cytometry (BD Biosciences, CA, USA) to determine the apoptosis rate.

### Statistical analysis

All experiments were repeated three times. Graph Pad Prism 5.0 was used for statistical analysis. A *t*-test or one-way analysis of variance followed by Tukey's post-hoc test was used to analyze the difference. Values of *p* < 0.05 were considered significant.

## Results

### Verification of Beclin-1 gene silencing efficiency

**Fig. 1:**
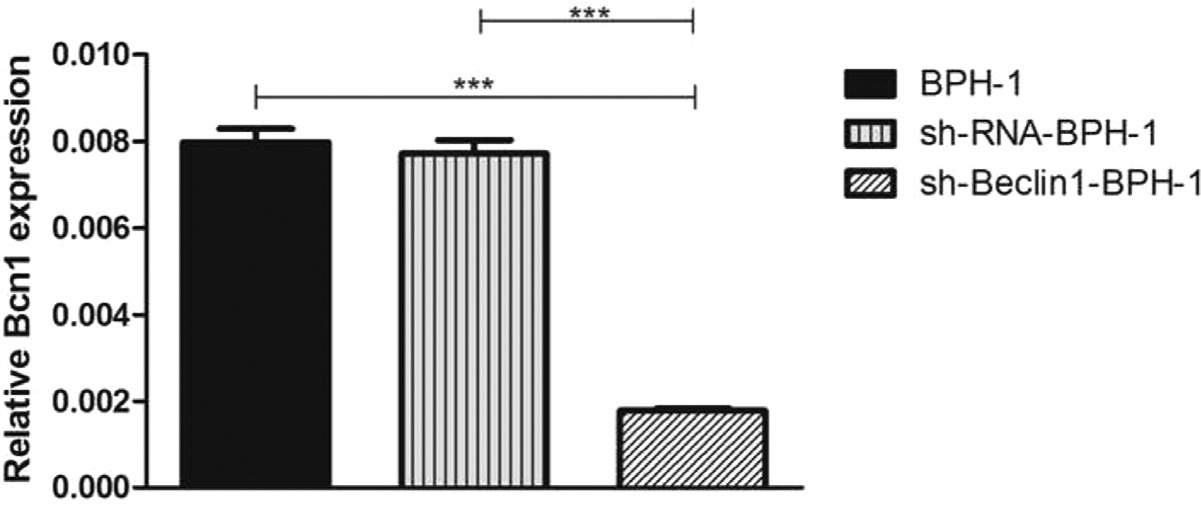
Efficiency of Beclin-1 silencing detected by RT-PCR. Data are representative of the relative expression of RNA that was normalized by the housekeeping gene GAPDH.

**Fig. 2:**
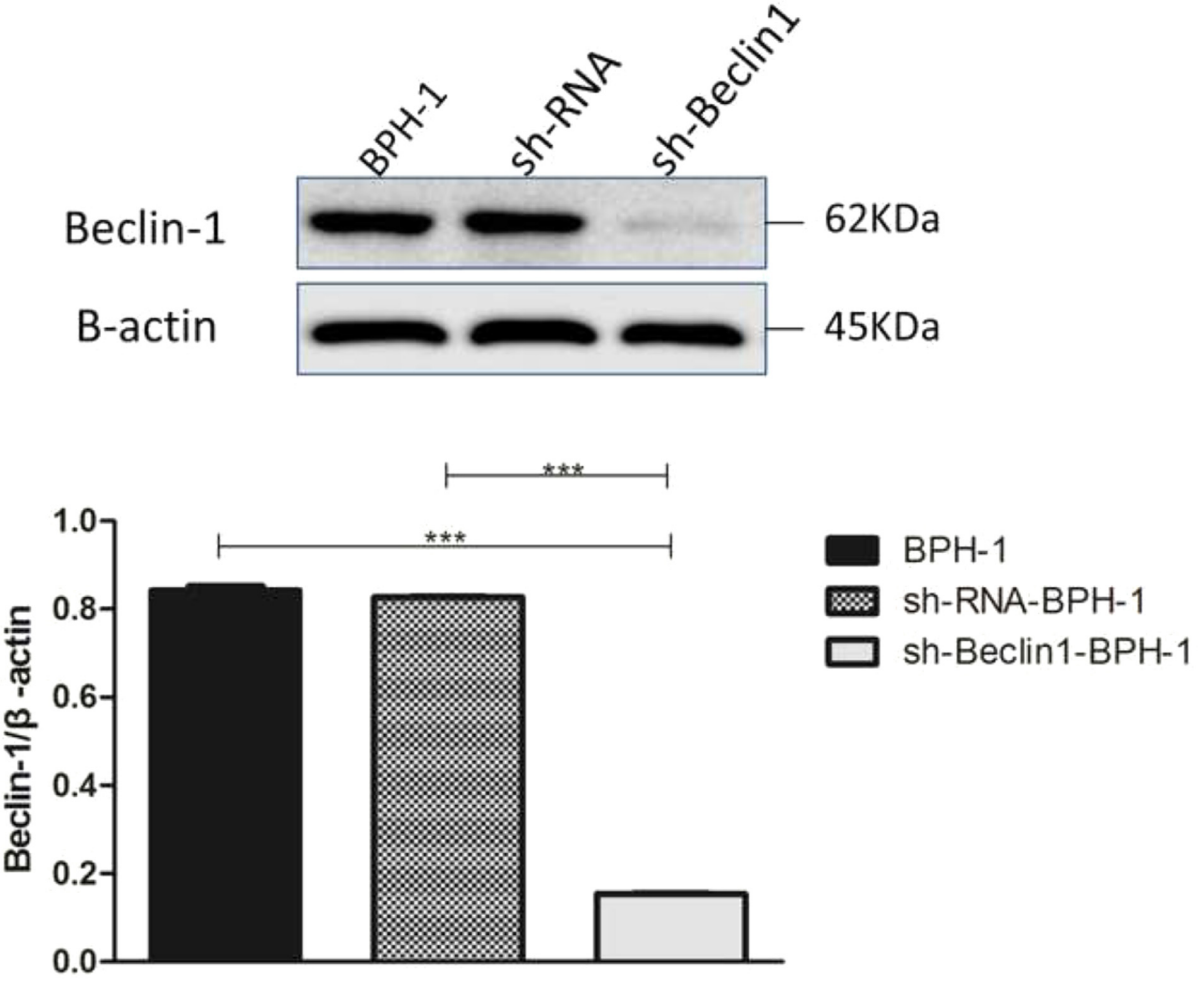
Efficiency of Beclin-1 silencing detected by Western blot. Data are representative of the relative expression of proteins that was normalized by β-actin.

After packaging, infection, and screening of lentivirus, the silencing efficiency of sh-Beclin1 was determined by RT-PCR (Fig. 1) and Western-blot (Fig. 2). There was no significant difference between the BPH-1 group and sh-RNA-BPH-1 group in the expression of Beclin-1 at both mRNA and protein levels (Figs. 1 and 2) (*p* > 0.05). The mRNA and protein expressions of Beclin-1 in the sh-Beclin1-BPH-1 group were significantly lower than those in the BPH-1 group (Figs. 1 and 2) (*p* < 0.001), suggesting that transfection of sh-Beclin1 can effectively silence Beclin-1 expression in BPH-1 cells.

### Beclin-1 silencing decreases free GFP fragments in GFP-LC3-expressing BPH-1 cells

**Fig. 3:**
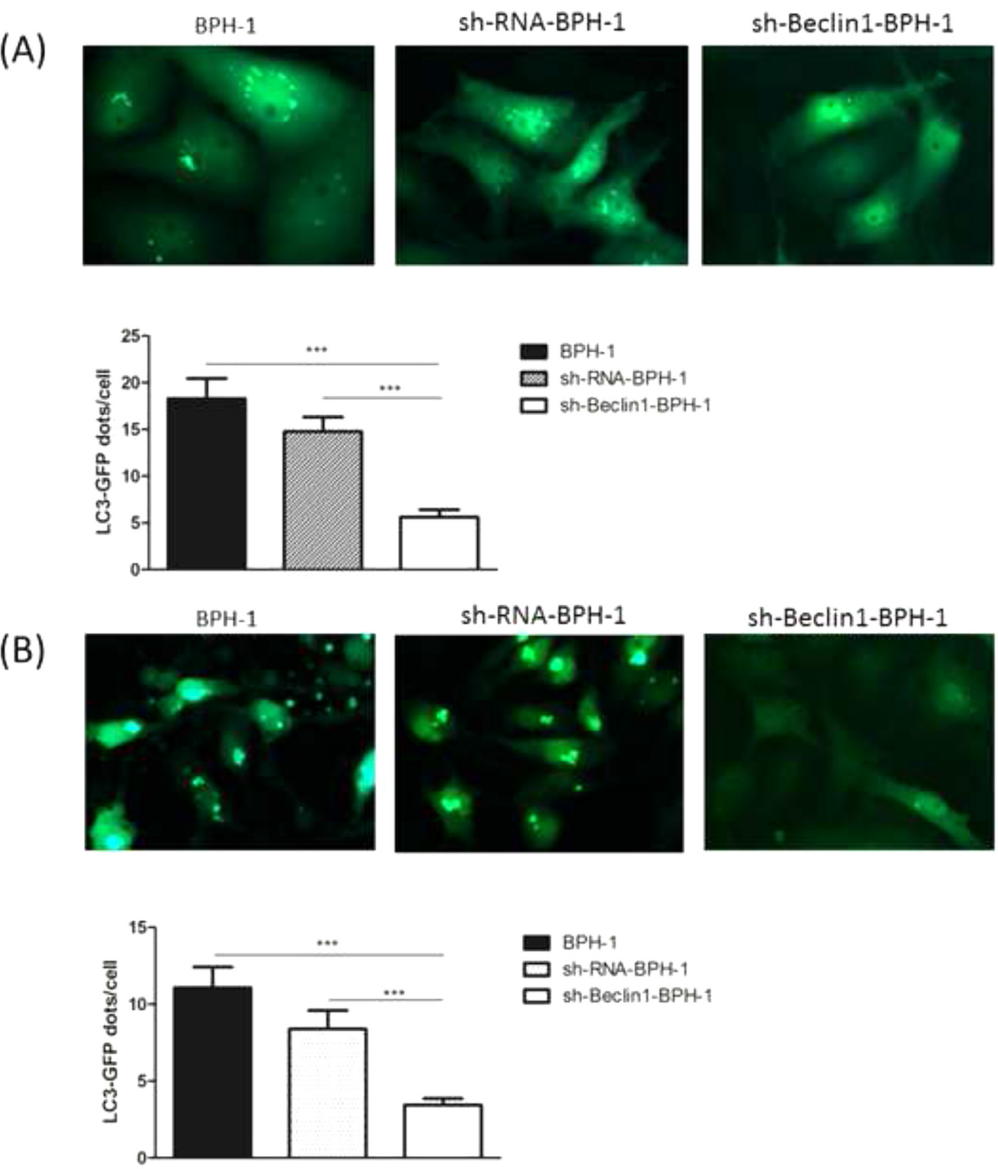
Silencing of Beclin-1 decreased the number of autophagosomes in BPH-1 cells. GFP-LC3 cleavage assay was carried out to monitor the autophagic flux. The expression of GFP-LC3 puncta was observed under different experimental conditions. (A) AD condition. (B) AD+AI condition.

It has been demonstrated that GFP-LC3 can behave similarly to endogenous LC3, while GFP is more resistant than LC3 to lysosomal degradation, resulting in increased free GFP fragments during autophagy induction.^25^ Hence, to trace the autophagic corpuscle in BPH-1 cells from different groups, GFP-LC3 adenovirus was transfected into BPH-1 cells before culturing in AD conditions combined with or without AI (AD or AD+AI). During AD, the number of GFP-LC3 puncta in BPH-1 cells was significantly decreased after knockdown of Beclin-1 (BPH-1 group vs. sh-Beclin1-BPH-1 group; *p* < 0.001) (Fig. 3A). Meantime, there were fewer GFP-LC3 puncta in the sh-Beclin1-BPH-1 group compared to the sh-RNA-BPH-1 group (*p* < 0.001) (Fig. 3A). Results of the assay under AD+AI conditions exhibited a similar tendency to those of the AD condition, indicating that the number of GFP-LC3 dots in the sh-Beclin1-BPH-1 group was significantly smaller compared to the BPH-1 and sh-RNA-BPH-1 groups (both *p* < 0.001) (Fig. 3B). Collectively, these results demonstrated that silencing of Beclin-1 could block the autophagy of BPH-1 cells under both AD and AD+AI conditions, suggesting the important role of Beclin-1 in the autophagy of BPH-1 cells.

### Beclin-1 silencing inhibits the expression of LC3-II protein in BPH-1 cells under AD+AI conditions

**Fig. 4:**
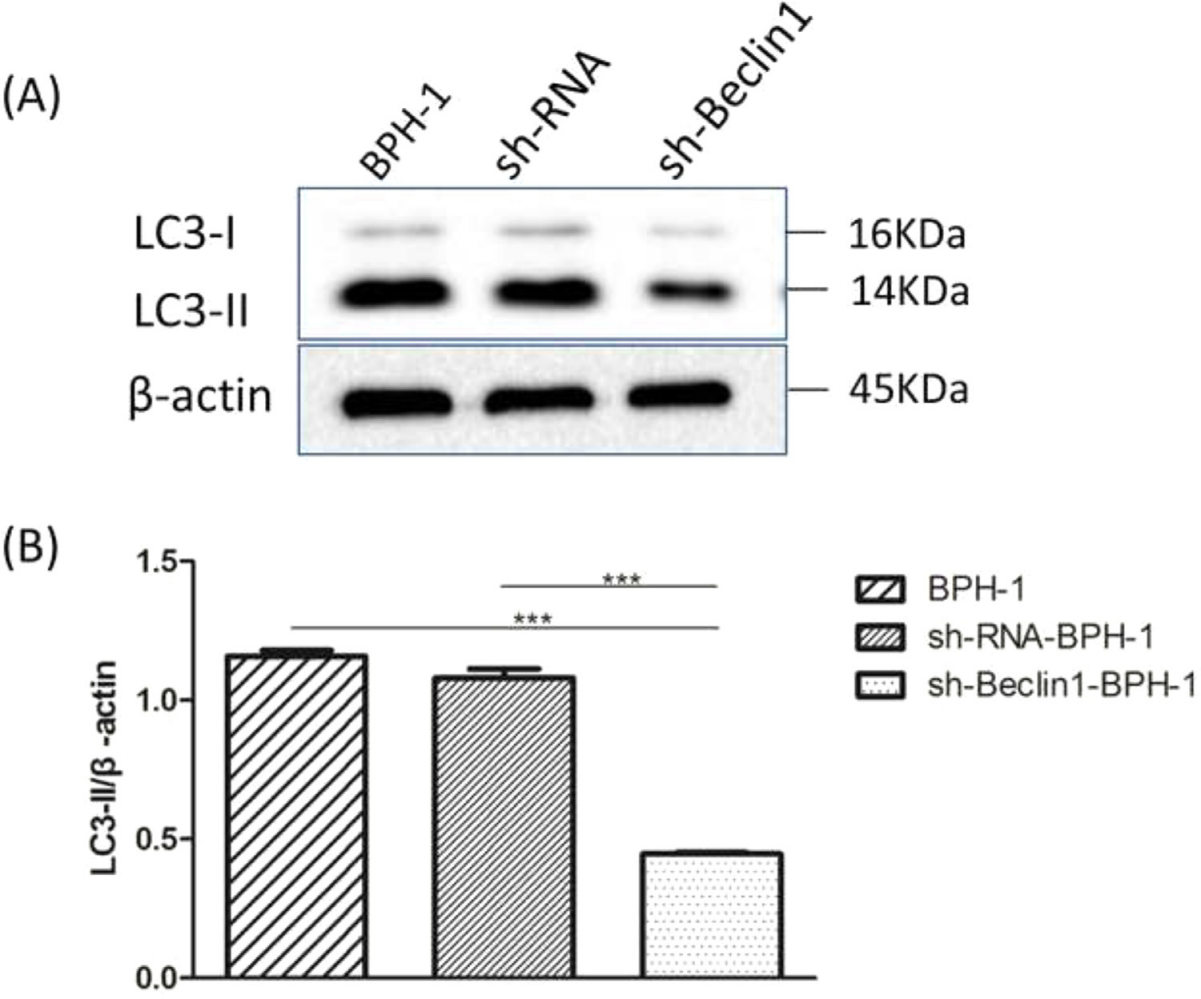
Silencing of Beclin-1 diminished the expression of LC3-II in BPH-1 cells. Cells in each group were cultured for 24 h under the AD+AI condition, and the expression of LC3 protein in each group was examined. (A) Protein bands of LC3 and β-actin. (B) The relative expression of LC3-II proteins that was normalized by β-actin.

It is well accepted that the conversion of soluble LC3-I to lipid-bound LC3-II is associated with the formation of autophagosomes. Therefore, Western blotting of LC3-II is another approach to investigate autophagy.^5^ Results showed that expression of LC3 II protein in the sh-Beclin1-BPH-1 group was obviously lower compared to the BPH-1 or sh-RNA-BPH-1 group (both *p* < 0.001) (Fig. 4), indicating that autophagy of BPH-1 cells could be attenuated by silencing Beclin-1.

### Beclin-1 silencing enhances apoptosis of BPH-1 cells under AD+AI conditions

**Fig. 5:**
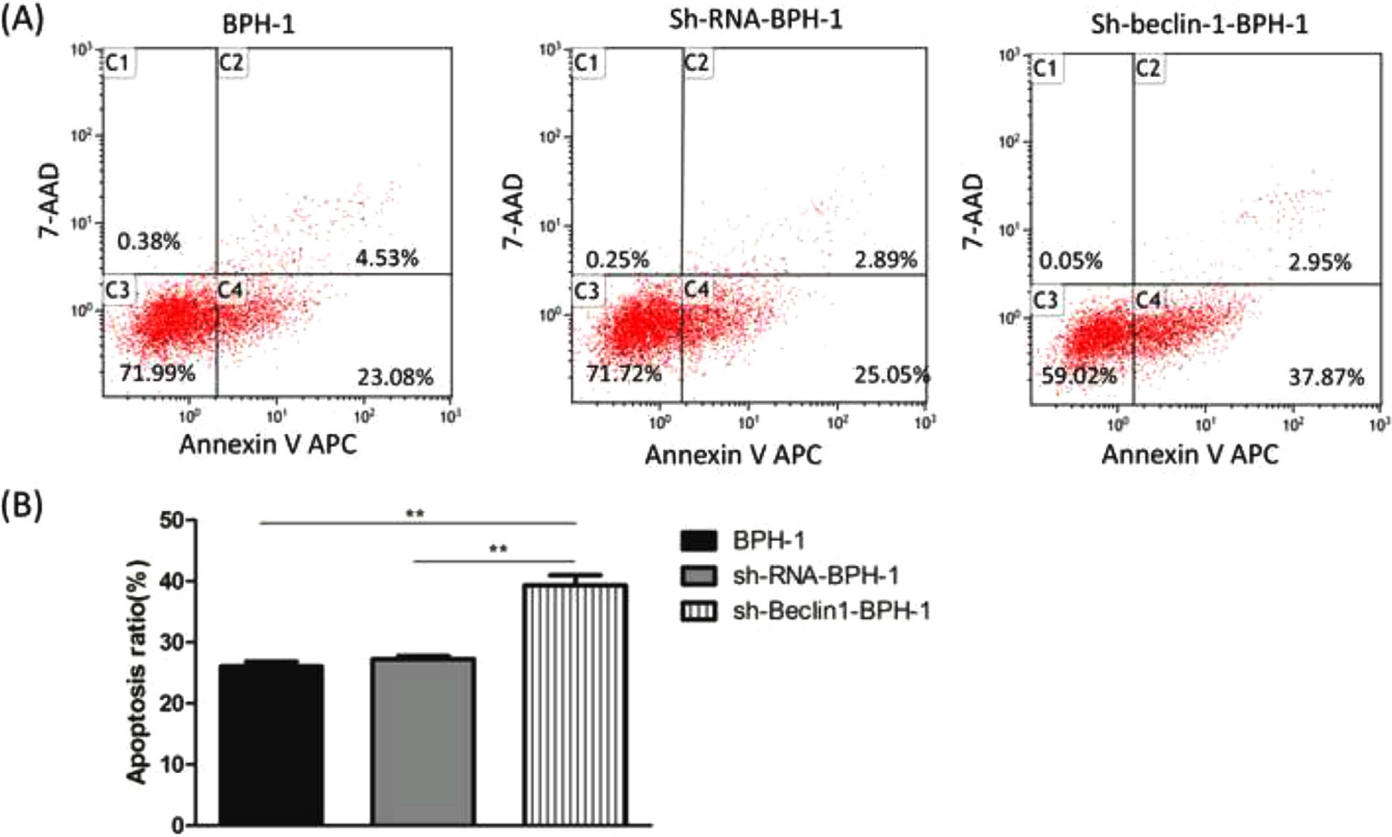
Silencing of Beclin-1 enhanced the apoptotic rate of BPH-1 cells. Cells in each group were cultured for 24 h under the AD+AI condition. (A) The apoptotic rate of cells from each group was detected by flow cytometry (Annexin V-APC/7-AAD). (B) The percentage of apoptotic cells was shown as the sum of annexin V-positive and annexin V/7-AAD double-positive cells.

Next, to explore the effect of Beclin-1 on the apoptosis of BPH-1 cells under the AD+AI condition, the apoptosis of BPH-1 cells was investigated using flow cytometry after silencing Beclin-1. The results showed that the apoptotic rate of the sh-beclin-1-BPH-1 group was significantly increased compared to the BPH-1 cell group or the Sh-RNA-BPH-1 group (both *p* < 0.01) (Fig. 5), suggesting that the signaling pathway-related apoptosis was activated after the Beclin-1 gene was silenced, which promoted apoptosis of the cells.

### The effect of Beclin-1 silencing on the expression of apoptosis-related proteins in BPH-1 cells under AD+AI condition

**Fig. 6:**
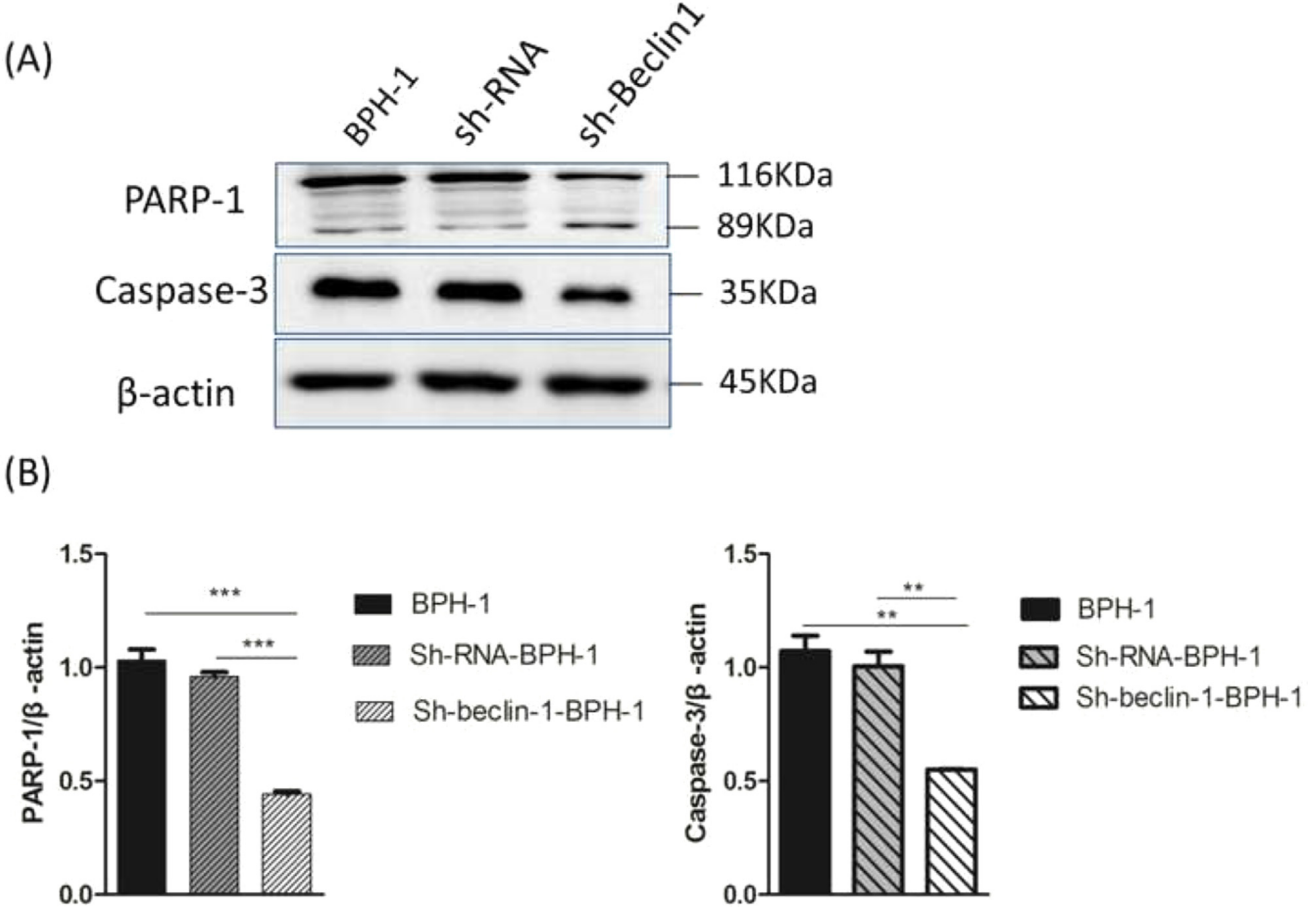
Silencing of Beclin-1 affected the activation of PARP-1 and Caspase-3 in BPH-1 cells. Cells in each group were cultured for 24 h under the AD+AI condition, and the expression of PARP-1and caspase-3 in each group was examined. (A) Protein samples of each group were detected by Western blot using anti-PARP-1 and anti-caspase-3 antibodies. (B) The relative expressions of PARP-1 and caspase-3 that were normalized by β-actin.

Apoptosis is a form of programmed cell death that is regulated by caspase and the Bcl-2 family of proteins. Therefore, the authors further examined the role of Beclin-1 in regulating the expression of apoptosis-related proteins. During apoptosis, Caspase-3 is activated universally to cleave several key proteins required for cellular function, including PARP-1. Western blot analysis showed that the expression of Caspase-3 in the sh-beclin1-BPH-1 group was obviously lower compared to the BPH-1 cell group or sh-RNA-BPH-1 group (both *p* < 0.01) (Fig. 6). Compared with the BPH-1 or sh-RNA-BPH-1 group, the expression of PARP-1 in the sh-Beclin1-BPH-1 group was significantly decreased (both *p* < 0.001), while the 89 kd fragmentation of PARP-1 was obviously increased (Fig. 6). These results indicated that Beclin-1 is involved in the activation of caspase-3 and cleavage of PARP-1.

**Fig. 7:**
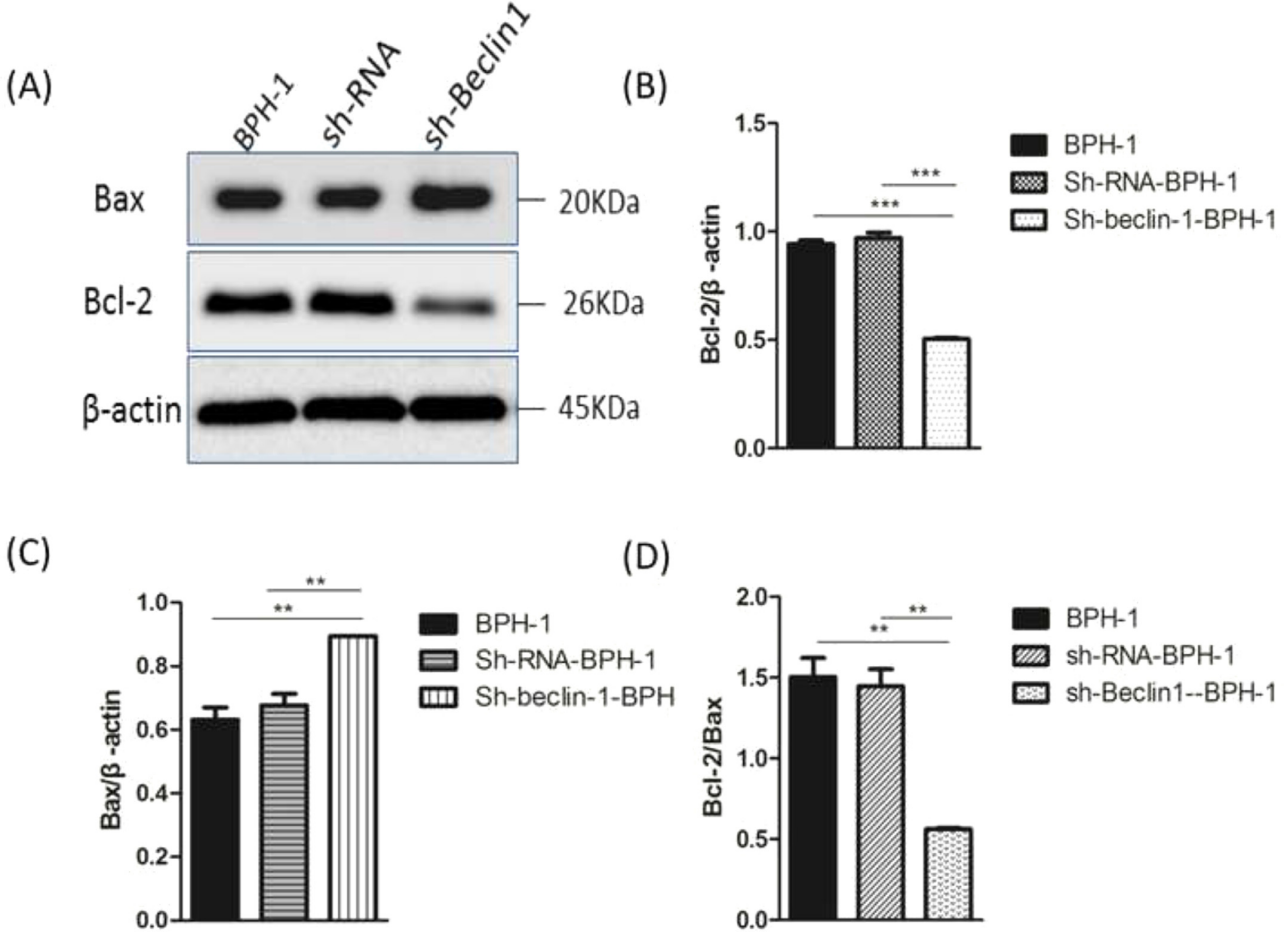
Silencing of Beclin-1 elevated Bax protein and lessen Bcl-2 protein expression BPH-1 cells. Cells in each group were cultured for 24 h under the AD+AI condition, and the expressions of Bax and Bcl-2 in each group were examined. (A) Protein samples were detected by Western blot using anti-Bax or anti-Bcl-2 antibodies. (B) The relative expressions of Bax and Bcl-2 proteins that were normalized by β-actin.

Bax and Bcl-2, two representative members of the Bcl-2 family, are broadly discussed pro- or anti-apoptotic proteins.^5^ In most cases, the proportion of Bcl-2/Bax determines apoptosis of the cells.^26^ Compared with BPH-1 or sh-RNA-BPH-1 groups, the expression of Bcl-2 in the sh-beclin1 BPH-1 cells was significantly reduced (both *p* < 0.001), while the expression of Bax was augmented (both *p* < 0.01) (Fig. 7). Moreover, the Bcl-2/Bax ratio of the sh-Beclin1-BPH-1 group was significantly lower compared to the other two groups. In summary, these results demonstrated that silencing of Beclin-1 aggravates the apoptosis of BPH-1 cells under the AD+AI condition, validating the results of flow cytometry.

## Discussion

In physiological conditions, cell growth in the organ depends on the dynamic balance between cell proliferation and apoptosis. Breaking this balance in the prostate gland will lead to BPH. Given that the most important characteristic of BPH has increased cell proliferation and reduced apoptosis,^27^ it is desirable to develop a therapeutic strategy that enhances apoptosis in BPH tissues. Androgens play a critical role in prostate development, growth, and pathogenesis. Inhibiting the action of androgen stimulates the apoptosis of prostate epithelial cells and facilitates pharmacological treatment of BPH.^28^ However, much evidence revealed that autophagy was induced during androgen inhibition and subsequently generated the “escaping effect” from apoptosis in prostatic epithelial cells, thereby restraining the efficiency of agents that can enhance apoptosis.^29^ Liu et al. showed that inhibiting autophagy enhanced AD-induced apoptosis in prostate epithelial cells.^14^ Therefore, understanding the mechanism underlying the crosstalk between apoptosis and autophagy is valuable for BPH treatment. The present study, for the first time, demonstrated that Beclin-1 plays a crucial role in the feedback loop between apoptosis and autophagy in BPH-1 cells.

In order to study the role of Beclin-1 in autophagy and apoptosis-related to BPH, sh-Beclin-1 plasmids were transfected into BPH-1 cells before performing a series of experiments. Initially, significant suppression of Beclin-1 was confirmed to occur at 48h in BPH-1 cells after sh-Beclin-1 transfection. Autophagy begins with the formation of autophagosomes. In this study, the GFP-LC3 cleavage assay demonstrated that once Beclin-1 was knocked down in BPH-1 cells, the number of autophagosomes was significantly reduced. This result demonstrated that Beclin-1 serves as a promoter of autophagy in BPH-1 cells under AD conditions. LC3 protein (30-kDa) could be cleaved into LC3-I protein that forms the autophagosome membrane and LC3-II. Thus, the autophagic activity could also be indicated by detecting the conversion of LC3-I to LC3-II by Western blot.^30^ The present data supported that silencing Beclin-1 significantly blocked the conversion of LC3-I to LC3-II, which further confirmed that the Beclin-1 gene was involved in the positive regulation of autophagy.

Several studies reported that the blockage of autophagy could lead to increased apoptosis in prostate carcinoma^13^ or benign prostate epithelial cells^31^ under AD conditions. Wirawan et al. identified Beclin-1 as a direct substrate of caspases, and the cleavage of Beclin-1 was observed in response to the mitochondrial pathways in apoptosis.^32^ Moreover, caspase-mediated cleavage of Beclin-1 has been proven to mediate the crosstalk between apoptosis and autophagy.^33^ In line with the above-mentioned findings, flow cytometry showed that the apoptotic rate of BPH-1 cells was markedly promoted by Beclin-1 silencing. This phenomenon might be explained by the Western-blot results that silencing of Beclin-1 boosted pro-apoptotic proteins and hampered anti-apoptotic proteins. Taken together, these results proved that silencing Beclin-1 was able to induce apoptosis of prostate epithelial cells more effectively under AD conditions, which was most likely associated with the blockage of crosstalk between apoptosis and autophagy-mediated by Beclin-1. However, the limitation of the current study is that it only focuses on the BPH-1 cell line, without an *in vivo* study to make the finding more credible. Hence, the present findings remain to be further verified by more studies of other prostate epithelial and stromal cells as well as *in vivo* BPH models.

In conclusion, Beclin-1 is a critical mediator of crosstalk between apoptosis and autophagy in BPH-1 cells. Hopefully, the present study's findings provide more scientific insight for future study regarding the possible application of autophagy in BPH treatment strategy under the AD condition.

## Funding

This work was supported by 10.13039/501100005270Fujian Science and Technology Project Plan, P.R.C (grant number 2018D0010).

## Authors’ contributions

Rongfu Liu and Song Zhang designed the research. Rongfu Liu, Song Zhang, Rui Wan, Jiang Deng and Wei Fang performed the experiments. Rongfu Liu drafted the manuscript.

## Declaration of Competing Interest

The authors declare no conflicts of interest.

## Appendix Supplementary materials

Image, application 1
