# Supplementary material for: Effect of Beclin-1 gene silencing on autophagy and apoptosis of the prostatic hyperplasia epithelial cells
Source: Clinics (Sao Paulo). 2022 Sep 8;77:100076. doi: 10.1016/j.clinsp.2022.100076 (PMC9468350; doi:10.1016/j.clinsp.2022.100076)
Supplement: Supplementary file 1 [file mmc1.docx]

**CLINICS-D-22-00038 – Supplementary Material**

**Table S1** The information of antibodies used in Western blot.

| **Antibody** | **Manufacturers** | **Cat.no** |
| --- | --- | --- |
| LC3 | Cell Signaling Technology | D3U4C |
| PARP-1 |  | 46D11 |
| Caspase-3 |  | 8G10 |
| Bax |  | D3R2M |
| Bcl-2 |  | C34C5 |
| Beclin-1 | BD Biosciences | 612112 |
| β-actin | Cell Signaling Technology | 13E5 |
| HRP-labeled secondary antibody | MultiSciences Biotech | GAR007 |
